# Targeting Persistent Human Papillomavirus Infection

**DOI:** 10.3390/v9080229

**Published:** 2017-08-18

**Authors:** Srinidhi Shanmugasundaram, Jianxin You

**Affiliations:** Department of Microbiology, Perelman School of Medicine, University of Pennsylvania, Philadelphia, PA 19104, USA; sshanmu@sas.upenn.edu

**Keywords:** HPV, persistent infection, cervical cancer, therapeutics, vaccines, episome maintenance, E2 protein

## Abstract

While the majority of Human papillomavirus (HPV) infections are transient and cleared within a couple of years following exposure, 10–20% of infections persist latently, leading to disease progression and, ultimately, various forms of invasive cancer. Despite the clinical efficiency of recently developed multivalent prophylactic HPV vaccines, these preventive measures are not effective against pre-existing infection. Additionally, considering that the burden associated with HPV is greatest in regions with limited access to preventative vaccination, the development of effective therapies targeting persistent infection remains imperative. This review discusses not only the mechanisms underlying persistent HPV infection, but also the promise of immunomodulatory therapeutic vaccines and small-molecular inhibitors, which aim to augment the host immune response against the viral infection as well as obstruct critical viral–host interactions.

## 1. Persistent HPV Infection

**Figure 1:**
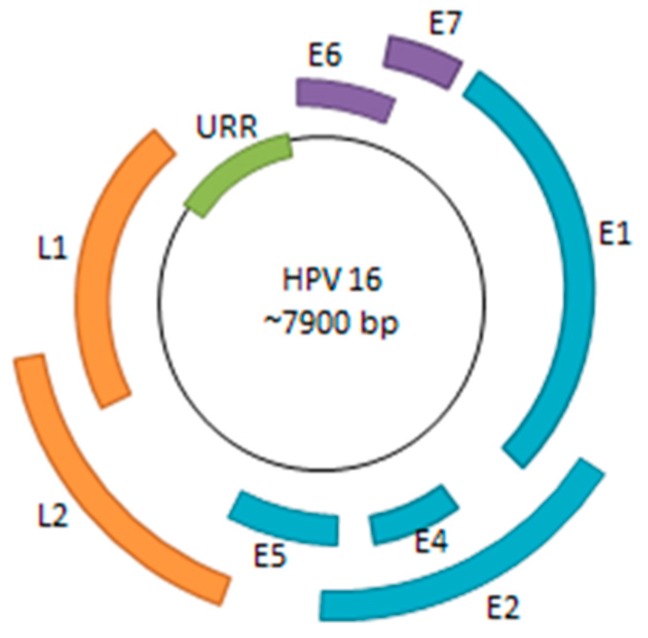
HPV Genome. The HPV genome consists of six early genes (*E1*, *E2*, *E4*, *E5*, *E6*, and *E7*) and two late genes (*L1* and *L2*). Many of the early genes are implicated in viral replication, transcriptional regulation, genome maintenance, along with immune system evasion. *E6* and *E7* are of particular interest as they are viral oncogenes that bind to and inactivate p53 and pRB, respectively. The URR (upstream regulatory region) consists of various promoter and enhancer elements as well as the viral origin of replication (ori).

Human papillomavirus (HPV) is a small, double-stranded DNA virus with a genome consisting of approximately 8000 base pairs. The HPV genome encodes six early genes (*E1*, *E2*, *E4*, *E5*, *E6*, and *E7*), two late genes (*L1* and *L2*), along with a non-coding region (Figure 1). Among the early genes, *E6* and *E7* are of particular significance due to their roles in inactivation of host tumor-suppressor genes and oncogenic progression [1]. The other early genes play critical roles in viral replication, transcriptional regulation, and viral genome maintenance—all necessary processes for sustaining persistent HPV infection [2].

**Figure 2:**
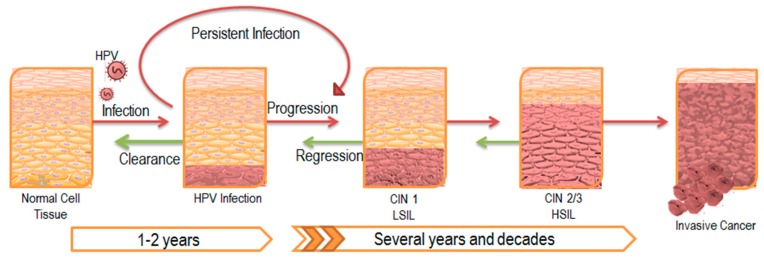
Progression of HPV Infection and Associated Disease. HPV typically establishes infection in the basal epithelial layer. A majority of these infections are transient and are cleared by the immune system within a couple of years. However, 10–20% of infections persist latently, leading to disease progression as illustrated by the red arrows. The lesion that develops as a result is also known as a central intraepithelial neoplasia (CIN) and is classified according to its severity. Eventually, low-grade squamous intraepithelial lesions (LSIL) advance to high-grade squamous intraepithelial lesions (HSIL), ultimately leading to invasive carcinoma. Despite tumor regression in response to initial treatment as illustrated by the green arrows, most cases of latent infection prevent complete clearance of the viral infection, and eventually results in lesion reoccurrence.

It has been established that persistent infection with HPV is associated with cervical, anogenital, as well as head and neck cancers [3,4]. In the majority of infected individuals, HPV infection is cleared by the immune system within a couple years of onset; however, the viral infection can continue to persist latently in a subset of the population (Figure 2). These patients with persistent HPV infection have an increased chance of acquiring epithelial cell abnormalities and subsequently developing cancers at the site of infection [5,6]. Though such progression to cancer is relatively rare, the prevalence of the virus among the general population makes HPV-associated persistent infection a statistically significant affliction.

Risk factors that may prevent the natural clearance of HPV persistent infection in certain populations have been a major source of interest. Several studies have discovered that genetic and lifestyle factors can significantly increase the probability of developing persistent infection [7,8]. For instance, multiple studies have found both smoking and alcohol use to be significant risk factors of persistent oral and genital HPV infection [7,9,10]. It has been proposed that the carcinogens in cigarette smoke increase viral load as well as the likelihood of cancerous transformation of the epithelial cells infected with HPV [11,12].

Interestingly, several genetic risk factors that predispose an individual to persistent HPV infection have also been identified, although the association is not particularly strong. The human leukocyte antigen (HLA) is one such genetic marker, of which certain alleles seem to have a more prominent association with an inability to clear HPV infection and the subsequent development of cervical cancer [13,14,15]. Given the variation in immunogenic profiles and associated risks among distinct ethnic groups, one study suggests that further investigation into these genetic markers for each population should be done in order to identify patients at an elevated risk for HPV persistence and to provide comprehensive preventative care accordingly [16].

Given the prevalence of infection with more than one type of HPV among patients, co-infection with multiple HPV types was investigated as a potential predictor of subsequent persistent infection. Results suggest that previous infection with HPV increases the chances of acquiring another HPV infection [17,18]. However, it is not definitive whether HPV persistence is dependent on co-infection [18,19]. Additionally, it has been found that variants within the specific HPV type may predispose an individual to persistent infection as well. For instance, one recent study discovered that three of the six HPV 16 E6 variants were associated with persistent infection; furthermore, of the nine HPV 16 E2 variants, two were linked to persistent infection [20]. While it is unclear at the time how these mutations mechanistically affect HPV persistence, it has been proposed that they may be linked to the virus’ ability to evade the immune system. In summary, studies seem to indicate that various risk factors may have contributing roles in HPV persistence among a small subset of infected patients [6,8,21]. However, additional investigation of the impact of these risk factors on the host immune system may paint a clearer picture of the development of persistent infection overall.

## 2. The Impact of Persistent Infection on Cancer

Approximately 95% of cervical cancer biopsies contain HPV viral genomes [1,22,23]. With cervical cancers being the second most common cancer among women globally, HPV is a significant infectious carcinogen that necessitates further investigation.

HPVs are classified into two major subcategories depending on the site of primary infection. Alpha HPVs generally infect genital epithelia and are further designated as high-risk and low-risk depending on their ability to induce cancer [24]. High-risk HPVs that are most frequently associated with malignant genital cancers include HPV 16, 18, 31, 33, and 45; on the contrary, low-risk HPVs, such as HPV 6 and 11, are mostly associated with benign papillomas at the site of infection [22,25]. Although more than one hundred types of HPV have been identified, two high-risk types, HPV 16 and HPV 18, are responsible for roughly 70% of cervical cancer cases [26].

In addition to being associated with cervical and anogenital cancers, persistent HPV infection has also been linked to head and neck cancers [4,27,28]. Several studies have additionally observed that women with cervical cancer had a greater risk of subsequently developing oral cancer [29]. These findings along with several similar studies established the presence of persistent HPV infection as a notable precursor to many genital as well as oropharyngeal cancers [3,30,31].

In cases of persistent HPV infection, an environment of genomic instability increases the likelihood of viral genome integration into the host genome [32]. In approximately 72% of cell samples taken from cervical carcinoma biopsies, HPV16 was integrated into the host genome [33,34]. However, the presence of carcinomas with solely episomal HPV and no detectable HPV integration implies that the progression to carcinoma does not necessitate integration of the papillomavirus genome [33]. Nonetheless, it is interesting to note that gene expression and DNA methylation patterns differ between cancers with integrated and non-integrated HPV genomes, suggesting that distinct oncogenic mechanisms may play a role in each setting [35].

A proposed consequence of viral genome integration that can result in oncogenic progression is the disruption of the *E2* open reading frame (ORF) [34]. Normally, the HPV E2 protein plays a critical role in regulating the activation and repression of viral promoters [36]. It was initially proposed that E2, by binding to sites proximal to the *E6*/*E7* promoter, is able to displace other transcriptional factors and thus prevent the formation of a transcription initiation complex [36,37]. The loss of *E2* expression is associated with the oncogenic progression of HPV as it consequently deregulates and increases the expression of viral oncogenic proteins, E6 and E7, which are known to disrupt tumor-suppressor genes *p53* and *pRB*, respectively [38,39]. It has also been shown that E6/E7 mRNA transcripts derived from integrated HPV 16 DNA display increased levels of stability, which in part contributes to the elevated steady-state levels of E6/E7 mRNA found in cervical cancers [40]. Integration of HPV may also contribute to cancer progression by interrupting the expression and function of key host cellular genes, thus promoting genomic instability [32,34]. Ultimately, it is clear that the progression to cancer from persistent infection is rather varied and can be affected by both the occurrence and location of integration.

## 3. Molecular Mechanisms Underlying Persistent HPV Infection

### 3.1. Viral Life Cycle and Immune System Evasion

After escaping the initial immune response, viruses must maintain their genomes within the host nucleus in order to achieve persistent infection. While integration into the host genome is an option favored by many chronic viruses, papillomaviruses like HPV maintain their genomes as extrachromosomal episomes that tether to host DNA [41]. By continuously replicating at low levels in a differentiating tissue, such as the basal epithelium, the papillomavirus is able to maintain a reserve in the host while simultaneously avoiding detection by the immune system [5,42]. During this stage of the infectious cycle, known as maintenance replication, viral genomes are able to partition themselves into the newly formed daughter cells by coordinating their replication with that of the host cell. Later in the infectious cycle, the virus enters a stage of vegetative amplification in which it replicates high levels of genomic products that are fated to be assembled into complete viral particles. This last stage tends to occur in terminally differentiating cell tissues, such as the upper epidermal layers that are destined to be sloughed off and thus are not strictly monitored by the host immune system. Higher levels of viral replication and assembly are observed in these layers because they tend not to trigger an immune response [42].

### 3.2. Viral Episome Hitchhiking on Host Mitotic Chromosomes

In order to ensure the viral genome is not lost during cell division, there is a tethering mechanism in place that attaches the viral genome to host mitotic chromosomes through protein intermediates [43]. Tethering viral genomes to mitotic chromosomes in dividing host cells is a common strategy used by many persistent DNA viruses, such as Epstein–Barr virus and Kaposi’s sarcoma-associated herpesvirus to name a few [44,45,46]. In the early stages of infection, the HPV E2 protein plays the major role in establishing persistence by tethering viral episomes to host mitotic chromosomes [47]. E2 is a multifunctional protein that is critical for supporting HPV infection. Its roles in viral transcription and replication have been extensively studied and reviewed [48,49]. In order to tether viral episomes to mitotic chromosomes, E2 binds to specific sites in the HPV episome using its C-terminal DNA-binding domain while interacting with chromosome-associated proteins through its N-terminal domain [50]. Unlike many cellular proteins which transiently bind chromosomes during mitosis, bovine papillomavirus (BPV) E2 was found to be bound to the chromosomes throughout all stages of mitosis [41]. Such a mechanism ensures that the viral genome is maintained in the nucleus of the daughter cell following cell division.

Interestingly, one study demonstrated that E2 protein encoded by HPV 11, 16 and 18 has the property to directly interact with the mitotic spindles, thereby maintaining HPV ori-containing DNA as “mini-chromosomes” in dividing cells. The likely region of HPV E2 involved in this mitotic spindle interaction is thought to be a 14-amino acid sequence that is highly divergent from its analogous sequence in BPV 1 E2 [51]. It is likewise important to note that, as indicated by the varying levels of viral genome copies per cell, segregation of the viral episomes is not an extremely specific process [43,47]. This suggests that viral genomes most likely associate randomly with host chromosomes and/or mitotic spindles during mitosis as tethered passengers. Since the levels of E2 also vary to a similar extent between cells, it is predicted that the E2 protein levels may correlate with the number of viral genome replicates in different cells [43]. Nevertheless, while it is well established that E2 is critical for episomal maintenance, it is not the sole player in episomal tethering. Interactions with host cellular proteins, such as those described below, are integral for maintaining persistent infection.

### 3.3. E2 Interaction with Host Receptors

Because of the critical role E2 plays in maintaining persistent infection, its interactions with host proteins are of particular interest. Though nearly all papillomaviruses express E2 and maintain their genomes through episomal tethering to mitotic chromatin, the host proteins targeted by the viral E2 protein are largely dependent upon the specific papillomavirus type [47].

An early proteomic study identified Bromodomain-containing protein 4 (BRD4), also known as mitotic chromosome-associated protein (MCAP), as a critical binding partner for E2 [52]. BRD4 is a member of a large family of proteins known as Bromo- and Extra-Terminal (BET) proteins which interact with acetylated histones in chromatin and function as “readers” of the histone code [53,54]. It is thought that BRD4 may have a “post-it note” function by associating with and marking specific segments of the mitotic chromatin in order to pass on epigenetic information to daughter cells. You et al. observed co-localization of E2 and BRD4 on condensed mitotic chromosomes and determined that the E2–BRD4 complex plays a role in BPV E2-mediated viral episome segregation [52]. X-ray crystallography experiments have clarified the specific binding between the N-terminal transactivation domain of HPV E2 and a highly conserved region of the C-terminal domain of BRD4 [55]. Furthermore, mutagenesis studies showed that deletions and mutations in the E2 N-terminal domain negatively impact E2–BRD4 binding as well as the chromosomal localization of E2 [56,57]. Additional studies have demonstrated that treating cells maintaining PV episomes with merely the C-terminal domain of BRD4 interferes with E2’s ability to tether viral episomes to mitotic chromosomes by competitively inhibiting its interaction with the functional full-length, chromosome-associated BRD4 [52,55]. Many, but not all, papillomaviruses seem to rely on BRD4 in order to maintain episomal tethering throughout mitosis [58].

In addition to episome maintenance, it has been demonstrated that BRD4 is vital for E2’s ability to activate transcription in all papillomaviruses [58,59,60]. Additional studies examining the E2–BRD4 interaction have shown that BRD4 may play a role in viral DNA replication as well [61,62,63]. In summary, the E2–BRD4 interaction plays a critical role in multiple stages of the HPV life cycle and therefore represents an excellent target for developing anti-viral therapeutics to terminate persistent infection.

The diversity within the papillomavirus clade supports the ability of its viruses to interact with various host cellular partners. Researchers have shown that α and β HPV E2 proteins likely interact with cellular targets in addition to BRD4 and furthermore, bind to distinct regions of the host chromosome [47,64]. In a study by Oliveira et al., E2 proteins from four α papillomaviruses have been shown to interact with host chromosomes in a temporally differential manner [47]. Possible factors that may contribute to the interaction between E2 and host chromatin include DNA helicase, TopBP1, and ChIR1 [64,65]. It was shown that HPV16 E2 co-localizes with TopBP1 possibly implying that TopBP1 is the cellular chromatin-associated receptor for E2 [66]. TopBP1 is a cellular protein closely regulated by the cell cycle, playing a role in initiation of cellular DNA replication, mitotic progression, as well as viral DNA replication. Experimentally, it has been demonstrated that the interaction between HPV16 E2 and TopBP1 contributes to viral DNA replication and episomal genome maintenance [64,67].

In addition to TopBP1, ChIR1 has been shown to be a key cellular partner of E2. Parish et al. demonstrated through intracellular co-localization experiments that HPV 11 and BPV 1 E2s co-localize with ChIR1 on the chromosome during prophase [65,68]. While ChIR1 migrates to the spindle poles following the transition to metaphase however, the E2 proteins from both viruses remain bound to the chromosome throughout mitosis. It has therefore been suggested that ChIR1 is essential for loading E2 onto mitotic chromosomes [65]. Since it has been shown that ChIR1 dissociates from the E2 mitotic foci at the conclusion of prophase, it is still likely that E2 relies on other host proteins such as TopBP1 or BRD4 to maintain its attachment to the chromosome; once again, the specific protein interactions may vary to an extent between papillomavirus types [47]. In summary, the E2–host protein interactions that support mechanisms of episome maintenance have revealed novel therapeutic targets for eliminating persistent infection.

## 4. Current Therapeutic Strategies for Clearing Persistent HPV Infection

### 4.1. HPV Prophylactic Vaccines

The development of prophylactic vaccines, such as Gardasil which targets HPV 6, 11, 16 and 18, marked a turning point in the HPV field as it provided an efficient preventative public health measure against HPV [21]. However, since immunological protection is limited to specific HPV types, Gardasil does not offer universal protection against HPV infection nor is it effective as a treatment for existing HPV infection. Nevertheless, because Gardasil proves highly effective against the most prevalent types of HPV, it has significantly reduced the overall statistical toll of the associated disease [69].

National two-dose vaccine programs remain both cost-effective and successful in preventing persistent HPV infection [70]. In recent years, a nine-valent HPV vaccine (HPV 6/11/16/18/31/33/45/52/58) that provides protection against approximately 90% of HPV-associated cancers and other diseases has been licensed by the FDA for use in young adults [71]. Despite the improving range and efficacy of these vaccines, HPV screening will remain a vital tool especially for at-risk women who have not been vaccinated or women in which the vaccine was infective [69,70,72]. It is also important to note that the HPV vaccines currently in use do not provide protection against all types of HPVs that are associated with cervical and other human cancers, thereby justifying the need for continual screening and development of additional therapeutic options to resolve cases post-infection.

### 4.2. The Promise of Therapeutic Vaccines

**Figure 3:**
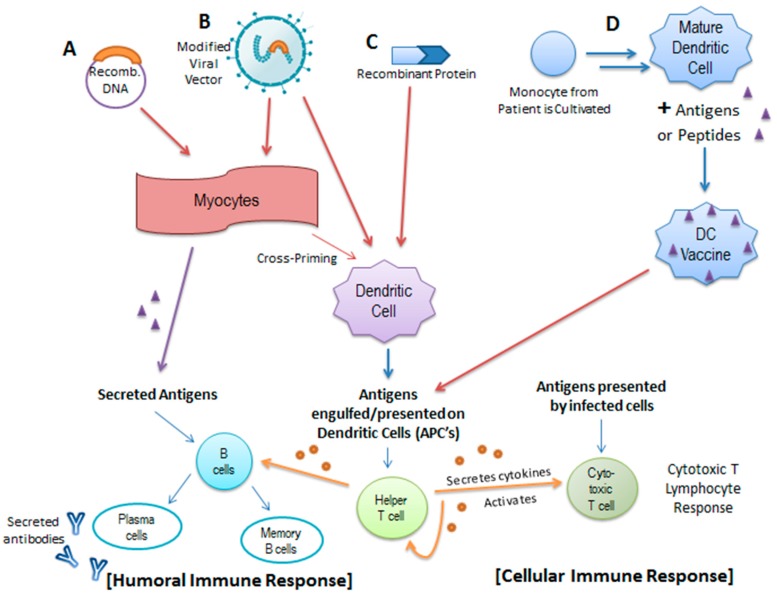
Activation of the Humoral and Cellular Immune Response by Various Forms of Therapeutic Vaccination. Therapeutic vaccines aim to strengthen and broaden the immune response to HPV by introducing specific antigens to a subset of antigen-presenting cells known as dendritic cells. (**A**) Recombinant DNA in the form of a plasmid encoding various antigens may be introduced intramuscularly to myocytes where they are transcribed and translated into antigen proteins, which are subsequently engulfed, processed, and presented by dendritic cells to downstream T-cells. The secreted antigens may also interact with B-cells to initiate a humoral immune response; (**B**) Recombinant DNA can be introduced in the form of a modified viral vector as well (i.e., Influenza A, Modified Vaccinia Ankara, etc.); (**C**) Additionally, the protein products can be introduced directly to the dendritic cells; in the case of HPV, recombinant protein vaccines usually consist of an E6/E7 fusion protein; (**D**) Dendritic whole-cell based vaccines are constructed from cultivated monocytes derived from the patients in combination with a particular antigen or peptide. These modified dendritic cells are then introduced to the patient’s immune system and similar humoral and cellular immune responses are incited through the activation of helper T-cells, B-cells, and cytotoxic T-cells.

Currently, clinical studies are also being conducted on potential therapeutic vaccines, which, unlike prophylactic vaccines, fight HPV infection via immunotherapy [73]. Most cases of HPV infection tend to be cleared by the immune system without intervention 1–2 years post-exposure; it is thought that persistent infection is most likely due to a lack of HPV-specific T-cell immunity [74]. Studies show that HPV-induced diseases indeed correlate with a weak HPV-specific CD4+ and CD8+ T-cell response [75]. Unlike prophylactic vaccines which rely on inducing specific antibodies and memory B-cells [76], therapeutic vaccines attempt to bolster HPV T-cell adaptive immunity. This is achieved through priming naïve T-cells to produce cytotoxic T lymphocytes (CTLs) that target HPV-infected cells, generating CD4+ T-cells to produce the necessary cytokines, and strengthening antigen-presenting cells (APCs). Dendritic cells are an important subset of APCs, which are involved in capturing and presenting antigens to T-cells and have been a central focus of many therapeutic vaccines [77,78] (Figure 3).

Numerous DNA-based vaccines have been developed to target persistent HPV infection and are currently in various stages of clinical study. These vaccines function by introducing a significant amount of viral DNA intradermally or intramuscularly to myocytes, which then express the antigen encoded by the DNA. The expressed, secreted antigen is then recognized and engulfed by dendritic cells and is subsequently expressed on MHC complexes, which are presented to CD8+ T-cells [77,78] (Figure 3A). GX-188E is one such recently developed therapeutic DNA vaccine that is designed to express E6 and E7 fusion proteins in order to increase the presentation of these HPV antigens by dendritic cells. The arrangement of the *E6* and *E7* genes within the DNA vaccine is shuffled to render the recombinant proteins incapable of degrading p53 and pRb [74]. GX-188E seems to be a promising therapeutic due to its demonstrated ability to induce an E6/E7-specific T-cell immune response in patients with high-grade lesions. Specifically, a polyfunctional CD8+ T-cell response was associated with clinical clearance of HPV as well as complete regression of lesions in seven of the nine patients who participated in the study [74]. However, despite the effectiveness demonstrated in this study, a greater sample size might be necessary to more thoroughly evaluate GX-188E’s response rate.

Electroporation, alongside DNA vaccination, has been shown to augment vaccine efficiency by modifying the cell membrane to increase DNA uptake and promoting inflammation and recruitment of APC’s at the site of vaccination [77]. In vitro studies have demonstrated that one method to stimulate a more effective HPV E6/E7 specific cytotoxic T lymphocyte response is to introduce HPV DNA through an oligomannose liposome (OML) as opposed to a standard liposome [79]. Further in vivo studies will be needed to evaluate the potential of OML-HPV to function as an effective therapeutic vaccine. Nevertheless, among the various classes of therapeutic vaccines, DNA- and protein-based HPV vaccines seem to be the less efficient, likely because they fail to produce a sufficient initial immune response [75,80] (Figure 3C). Adjuvants, such as imiquimod or cidofavir, which serve as agonists to various toll-like receptors, are necessary alongside these vaccines to augment the initial immune response to the vaccine and imbue long-lasting protection [73].

To counter this problem of low vaccine efficacy, new models of therapeutic vaccines have been proposed. For instance, a recent study utilizing live attenuated influenza A virus as the vaccine vector for the expression of HPV E6–E7 fusion transgenes demonstrated its ability to elicit a broader immune response relative to vaccination with the recombinant protein alone [81]. In this case, the influenza vector itself functions as an adjuvant (Figure 3B). This study reveals the potential of this recombinant vaccine not only to induce a stronger cellular immune response but also to promote lesion regression in mice. Additionally, because no DNA intermediates are created during the influenza life cycle, any theoretical integration of E6 or E7 into the host genome is elegantly prevented, providing an additional measure of safety [81].

Clinical studies have demonstrated the potential of utilizing Modified Vaccinia Ankara (MVA) as an alternative attenuated viral vector for designing therapeutic vaccines as well. Recombinant MVA engineered to express BPV E2 (MVA E2) has demonstrated its potential to serve as an alternative intralesional treatment for HPV-induced lesions [82,83]. Following a phase II clinical trial involving 34 patients with high grade lesions, it was observed that MVA E2 promoted a specific cytotoxic response in all patients, as demonstrated by the generation of antibodies specific to MVA E2 [82,83,84]. Additionally, treatment with MVA E2 resulted in elimination of lesion in 58.9% of patients and significant reduction (up to 60%) of lesion size in 41.2% of patients [84]. Unlike control patients who were treated with conization, patients receiving MVA E2 did not show signs of lesion recurrence [84]. These results illustrate that physical removal of the lesion, though temporarily effective, is not a permanent method to eliminate persistent basal infection. The efficient elimination of lesions, prevention of recurrence, along with the absence of adverse side effects, make MVA E2 and similar therapeutic vaccines potentially strong candidates in the future of HPV therapeutics.

Dendritic whole-cell based (DC) vaccines have also been proposed as a therapeutic strategy for patients with early stages of cervical cancer (Figure 3D). These vaccines are developed by culturing patient-derived monocytes into mature dendritic cells followed by pulsing with a particular antigen or peptide. When these modified dendritic cells are then introduced to the patient, a similar humoral and cellular immune responses is incited through the activation of downstream immune processes [85]. Clinical studies of full-length HPV E7-pulsed dendritic cell vaccines have demonstrated the tolerability of DC vaccines as well as an E7-specific antibody response in all immunized patients, although the observed T-cell response was somewhat variable among the patients tested [86].

Despite the advances made, there are significant challenges in the development of therapeutic vaccines [87]. Many forms of vaccines, though promising in vitro and in vivo, have shown to be ineffective clinically, possibly because the induced CD4+ and CD8+ T cell responses were neither strong enough nor broad enough [74,75]. Several of these challenges stem from HPV persistent infection triggering a series of events that downregulate the immune system [69,75,80]. Currently, studies concerned with immune-suppressive mechanisms involving T regulatory cells are being conducted, which may lead to suitable therapeutics that promote a more favorable balance of immune reactions [69,75,80]. Given these findings, the most pragmatic approach to achieve complete clearance may be through a combination of antiviral and immunomodulatory treatments.

### 4.3. Chemopreventive Strategies

An interesting pilot study has found that an intra-vaginal infusion of CIZAR^®^, a zinc-citrate compound, was effective in eliminating several types of cervical high-risk HPV infection [88]. Although the mechanism by which zinc operates to bring about this effect has not yet been thoroughly investigated, it is thought that the zinc component activates a cellular immune response by inducing T-cells [88]. Further studies will need to be conducted to conclusively characterize the mechanisms by which this zinc-citrate compound functions and determine its efficacy towards clearance of HPV infection and prevention of lesion recurrence.

### 4.4. Small Molecular Inhibitors

As may be inferred from previous discussion, protein–protein interactions provide more specific therapeutic windows to clearing persistent HPV replication. By interrupting processes critical to the HPV life cycle such as DNA replication, episome maintenance, or viral transcription, latent infection may be terminated because the virus is unable to maintain its genome in dividing cells. Small molecular inhibitors and nucleic acid-based treatments that target these interactions have shown to be promising non-conventional therapeutics and are currently undergoing clinical trial [89]. However, targeting specific protein–protein interactions will also be heavily dependent on the stage of cancer progression as well as the papillomavirus type. Therefore, in addition to advancing therapeutic options, specific diagnostic and classification systems of HPV disease progression also need to be developed.

The interaction between E2 and its host binding partners is a strong prospective target for therapeutic development because of its role in episomal maintenance, as well as viral transcription and replication. For example, blocking the BPV E2–BRD4 interaction prevents mitotic chromosomal localization of E2 and viral genome, thereby preventing episomal maintenance [52]. In later studies, a similar result was also found to occur as a result of inhibiting HPV 16 E2–BRD4 interaction [57]. Moreover, recent studies have demonstrated that interruption of E2–BRD4 function can additionally inhibit viral gene expression and replication [58,60,61]. Additional studies illustrate that targeting the interaction between E2 and other identified host cellular proteins could be an efficient therapeutic strategy to eliminate persistent infection [47,48,64,65,66], providing a framework with which to approach developing HPV-specific therapeutics. Highly specific visualization/detection techniques such as bimolecular fluorescence complementation and Mammalian Protein–Protein Interaction Trap (MAPPIT) will likewise prove integral to identifying these synthetic and naturally occurring small molecule inhibitors [57,90,91,92].

Nevertheless, it is crucial to consider that therapeutics targeting E2 and host interactions may prove only to be relevant in the stages of HPV infection in which integration has yet to occur in order to eliminate episomal HPV. Although proposed E1–E2 inhibitors have the same theoretical effect in terms of inhibiting persistent infection by preventing viral replication, the nuanced variations in E1–E2 structural binding between papillomavirus genera make it a difficult therapeutic target [49]. Due to the divergence of amino acid sequences among HPV proteins of different types, realistically, various classes of drugs will likely be necessary to interfere with multiple specific viral–host interactions.

## 5. Perspectives and Future Direction

While the advancement of Gardasil and other multivalent prophylactic vaccines provide preventative public health measures against HPV infection, the burden associated with HPV remains high in regions where access to regular screening and vaccination are unavailable. Therefore, the development of antiviral agents along with therapeutic vaccines to treat HPV post-infection remains imperative.

Interrupting virus-host interactions critical to HPV persistence demonstrates great potential to terminate persistent HPV infection, which may otherwise proceed to malignant cancer. Notably, these protein–protein interactions are relatively divergent between PV types and miniscule changes in relevant target protein amino acid sequences can drastically affect drug efficiency. Therefore, the likelihood of developing a pan-HPV antiviral currently remains low. Targeting the better-conserved cellular proteins with which HPV proteins interact provides an alternative option for developing a pan-HPV therapeutic; however, the deleterious effects caused by interrupting these proteins during normal cellular processes may outweigh the potential benefits derived from interfering with viral function. Specific drug delivery measures and research into the comprehensive cellular pathways associated with HPV pathogenesis may minimize these unfavorable outcomes. Practically speaking, multiple classes of antiviral drugs may be necessary in order to efficiently treat persistent HPV infection based on HPV types, stages of infection, and sites of infection. Despite these reservations, insights into the molecular mechanisms of HPV pathogenesis revealed in the last decade hold promise for the development of highly specific and effective antiviral agents and immunomodulatory vaccines for treating HPV persistent infection.
